# Supplementary material for: High Oxygen Does Not Increase Reperfusion Injury Assessed with Lipid Peroxidation Biomarkers after Cardiac Arrest: A Post Hoc Analysis of the COMACARE Trial
Source: J Clin Med. 2021 Sep 17;10(18):4226. doi: 10.3390/jcm10184226 (PMC8471647; doi:10.3390/jcm10184226)
Supplement: Supplementary file 1 [file jcm-10-04226-s001.zip › jcm-1365945-Supplementary.pdf]

Journal of
Clinical Medicine

MDPI

SUPPLEMENTAL MATERIAL

High oxygen does not increase reperfusion injury assessed with lipid peroxidation biomarkers after cardiac arrest: a post hoc analysis of the COMACARE trial

Jaana J Humaloja, Maximo Vento, Julia Kuligowski, Sture Andersson, José D. Piñeiro-Ramos, Ángel Sanchez-Illana, Erik Litonius, Pekka Jakkula, Johanna Hästbacka, Stepani Bendel, Marjaana Tiainen, Matti Reinikainen, Markus B. Skrifvars, COMACARE study group

Content

Study Analysis Protocol and Statistical Methods 1
Method for Biomarker Determination 4
Fig S1 6
Fig S2 7
Fig S3 7
Table S 1 8
Fig S4 8
Fig S5 9
Table S2 12
Table S3 13
Table S4 14

Study Analysis Protocol and Statistical Methods in Detail

Trial design, participants, and inclusion criteria

This study is based on post hoc analysis of arterial blood samples collected during COMACARE-trial (NCT02698917). The complete trial protocol and main findings have been published previously in detail [1]. Shortly, we prospectively randomized 123 patients resuscitated from out of hospital cardiac arrest (OHCA) with 2^3 factorial design to normal or moderately elevated arterial oxygen tension (PaO_2 10-15 kPa or 20-25 kPa), low-normal or high-normal arterial carbon dioxide tension ($PaCO_2$ 4.5 -4.7 kPa or 5.8-6.0 kPa), and low-normal or high-normal mean arterial pressure (65-75 mmHg or 80-100 mmHg) for the first 36 hours of the intensive care unit (ICU) treatment. The randomization was done upon ICU admission, the application of treatment targets was initiated simultaneously (later referred as time-point 0h). All patients were treated according to the current guidelines and all patients underwent targeted temperature therapy (TTM) 33 °C or 36 °C. Participants included adult (age of 18 to 80) patients resuscitated from witnessed out-of-hospital cardiac arrest, whose initial rhythm was ventricular fibrillation or ventricular tachycardia, who had markedly impaired level of consciousness (defined as no response to verbal commands and Glasgow coma scale motor score < 5 (withdrawal to painful stimuli at best)), and who were mechanically ventilated upon ICU admission. Patients in this sub study of COMACARE trial are from six different ICUs in Finland. Flow chart of patient inclusion can be found later in this online supplemental material, Figure E1.

Blood samples and sample analysis

Arterial blood samples for biomarker determination were collected from March 2016 until November 2018, and the samples were analyzed in May 2020. We determined the relative levels of isoprostanes, isofurans, neuroprostanes, neurofurans, di-homo-isoprostanes, and di-homo-isofurans from samples collected on ICU admission (0 h) and 24, 48,

and 72 hours after admission. The samples were analyzed in the Analytical Unit, Health Research Institute La Fe, Valencia, Spain, employing ultra-performance liquid chromatography tandem mass spectrometry (UPLC-MS/MS). [2,3] More specified description of the analysis method used can be found further in this online supplemental material.

Outcome measures

The primary outcome measures were lipid peroxidation biomarker levels (IsoPs, IsoFs, NeuroPs, NeuroFs, Di-homo-IsoPs, Di-homo-IsoFs) at 0 and 24 hours. A secondary outcome used in two sub analyses was patient neurological outcome after 6 months of the arrest determined with cerebral performance category by an experienced neurologist blinded to study group allocation [4]. Favorable functional outcome was defined as a Cerebral Performance Category score of 1 or 2, corresponding to independence in activities in daily life as a minimum, and unfavorable outcome as Cerebral Performance Category score of 3 to 5, corresponding to a spectrum from severe cerebral disability to death.

Oxygen Exposure

Oxygen exposure was measured in three settings: prehospitally, on ICU admission, and during the first 24 hours of ICU care. We obtained prehospital PaO_2 from the first arterial sample collected after return of spontaneous circulation (ROSC). On ICU admission oxygenation was determined from the first PaO_2 measured after arrival to the ICU and simultaneously used FiO_2. Finally, we determined the highest PaO_2, PaO_2 area under the curve, and FiO_2 area under the curve over the first 24 hours of the ICU treatment.

Data analysis protocol

First, we compared the first measured prehospital PaO_2 after return of spontaneous circulation with biomarker levels on ICU admission in scatterplot. Second, we compared on ICU admission measured PaO_2 and used FiO_2 with biomarker levels on ICU admission in scatterplot. We visualized biomarker levels at 24 hours after ICU admission in scatterplots with the highest PaO_2, PaO_2 area under the curve, and FiO_2 area under the curve over the first 24 hours of the ICU treatment and compared the biomarker levels between the two randomized oxygen target groups (PaO_2 10-15 kPa vs 20-25 kPa). Additionally, we compared time from the arrest to the ICU admission with biomarker levels at 0 h in scatterplots. Third, we demonstrated a time-profile for different lipid peroxidation biomarker levels at 0, 24, 48 and 72 hours. We compared biomarker levels over time between patients with different randomized PaO_2 target and between patients with favorable vs unfavorable functional outcome. Finally, we built a linear regression models to predict biomarker levels at 0 and 24 hours.

Statistical methods

We present continuous data as medians and interquartile ranges (IQR) and categorical data as counts and percentages. We tested all continuous variables for normality and used Mann-Whitney U test to compare non-normally distributed data [5]. We compared categorical data with Pearson's chi squared test [6]. Association between oxygen exposure and biomarker levels were analyzed with scatterplot visualizations, linear regression, and linear mixed model analysis [7,8]. We performed Lg-transformation for values of biomarker concentrations to stabilize variation and to make skewed data normal. Areas under the curve for oxygen exposure were computed using the trapezoid rule [9]. We used linear mixed model to compare biomarker levels over time [7]. Linear regression models for biomarker levels at 0 hours included the PaO_2 and FiO_2 values on ICU admission, and a computed propensity score for high (higher than the median) admission PaO_2 as confounding factors [10]. Propensity score for high admission PaO_2 was used to simplify several confounding factors as one variable in the model. We computed the propensity score with binomial logistic regression with patient age, gender, body mass index, delay to first

unit, and time from the arrest to return of spontaneous circulation time, and we chose the variables from the univariate analyses with p-value < 0.20 based on analysis of patient baseline characteristics shown in the manuscript in Table 1 [11]. Linear regression models for biomarker levels at 24 hours included patient age, presence of bystander cardiopulmonary resuscitation (yes/no), and original randomization groups for PaO_2, $PaCO_2$ and mean arterial pressure as confounding factors. We selected variables for linear regression with biomarker levels at 24 h after clinical consideration and by testing the model accuracy with multiple parameters. Statistical analyses were performed with IBM SPSS statistics for macOS version 27.0.1.0 and Graph Pad Prism version 9.0.1. for macOS, and graphical illustrations are conducted with Graph Pad Prism version 9.0.1 for macOS.

Method for Biomarker Determination

Standards and reagents

Potassium hydroxide (KOH) was purchased from Sigma Aldrich Quimica S.A. (Madrid, Spain), methanol (MeOH), n-heptane, and acetonitrile at LC-MS grade were purchased from J.T. Baker (Phillipsburg, NJ, USA), and formic acid (98% w/w) and ethyl acetate (analytical grade) were from Panreac (Barcelona, Spain). Ultrapure H_2O generated on a Milli-Q® integral system from Merck Millipore (Darmstadt, Germany) was used. Deuterated prostaglandin $F_{2\alpha}$ ($PGF_{2\alpha}$-D_4) with a purity of ≥98% and a deuterated incorporation of ≥99% was purchased from Cayman Chemical Company (Ann Arbor, MI, USA) and used as an internal standard (IS).

Sample preprocessing

Samples were processed following previously described methods [2,3]. Plasma was thawed on ice and 100 μL of KOH (15% w/v) were added to 100 μL of each sample followed by incubation for 30 min with gentle agitation at 40 ^{0}C in a WSB-18 water bath from Witeg (Wertheim, Germany). Then, samples were diluted with 700 μL of H_2O:CH_3OH:HCOOH (82.6:14.6:2.8 v/v) containing 100 nM of IS, homogenized on a Vortex mixer for 5 s and kept on ice for 10 min. Samples were centrifuged at 16000 x g for 10 min at 4 ^{0}C and supernatants were collected. Solid phase extraction (SPE) employing Discovery® DSC-18 SPE 96-well plates was carried out with a negative pressure manifold, both from Sigma-Aldrich (St. Louis, MO, USA). SPE cartridges were conditioned with 1 mL CH_3OH followed by 1 mL H_2O (0.1% v/v HCOOH, pH 3) and samples were loaded into the SPE wells. Each well was washed with 1 mL H_2O (0.1% v/v HCOOH, pH 3) and 500 μL heptane. Cartridges were dried with negative pressure and analytets were eluted with 4 x 100 μL ethyl acetate. Sample extracts were evaporated using a miVac centrifugal vacuum concentrator from Genevac LTD (Ipswich, UK) and dissolved in 60 μL of H_2O (0.1% v/v HCOOH, pH 3):CH_3OH (85:15 v/v).

Ultra Performance Liquid Chromatography – tandem Mass Spectrometry (UPLC-MS/MS) analysis

UPLC-MS/MS analysis was carried out employing an Acquity-Xevo TQS system from Waters (Milford, MA, USA) operating in the negative electrospray ionization (ESI^-) mode. Instrumental conditions were selected as follows: capillary voltage was set to 2.9 kV, source temperature was 150 ^{0}C, desolvation temperature was 395 ^{0}C, and nitrogen cone and desolvation gas flows were set to 150 and 800 L h^{-1}, respectively. Dwell time was set to ensure a minimum of 10 data points per peak. Separation conditions were selected to achieve appropriate chromatographic retention on a Waters BEH C18 reversed phase column (2.1 x 100 mm, 1.7 μm) using a H_2O (0.1% v/v HCOOH):CH_3CN (0.1% v/v HCOOH) binary gradient. Flow rate, column temperature and injection volume were set to 450 mL min^{-1}, 45 ^{0}C, and 9 μL, respectively. The gradient with a total run time of 7.0 min was as follows: from 0.0 to 0.1 min 15% v/v CH_3CN (0.1% v/v HCOOH) (i.e., channel

B); from 0.1 to 5.0 min %B increased up to 40%; from 5.0 to 6.0 min %B increased up to 75%; between 6.0 and 6.15 conditions were held constant at 75% B followed by the return to initial conditions (i.e., 15% B) between 6.15 and 6.25 min; conditions were maintained for 0.75 min for system re-equilibration. MS detection was carried out by multiple reaction monitoring (MRM) employing the acquisition parameters shown in Table S1. Relative signal intensities normalized using the IS signal were determined. Total parameters were considered when the area was >3 times the signal of a blank. The UPLC-MS/MS system was operated employing MassLynx software version 4.0 from Waters.

Acquisition parameters employed for the determination of isoprostanoids.

Compound family	m/z parent ion	Cone [V]	CE [eV]	m/z daughter ion	RT (min)	Internal Standard
Isoprostanes (IsoPs)	353.20	35	30	115.00	3.5 – 6.0	$PGF_{2\alpha}$-D_4
Isofurans (IsoFs)	369.20	45	20	115.00	1.9 – 6.6	$PGF_{2\alpha}$-D_4
Neuroprostanes (NeuroPs)	377.20	35	20	101.00	2.0 – 6.0	$PGF_{2\alpha}$-D_4
Neurofurans (NeuroFs)	393.00	35	20	193.00	1.9 – 6.6	$PGF_{2\alpha}$-D_4
Dihomo-Isoprostanes (Dihomo-IsoPs)	381.00	20	20	143.00	5.3 – 6.7	$PGF_{2\alpha}$-D_4
Dihomo-Isofurans (Dihomo-IsoFs)	397.00	35	25	155.00	3.4 – 6.6	$PGF_{2\alpha}$-D_4
$PGF_{2\alpha}$-D_4	357.00	40	30	197.00	4.9	-

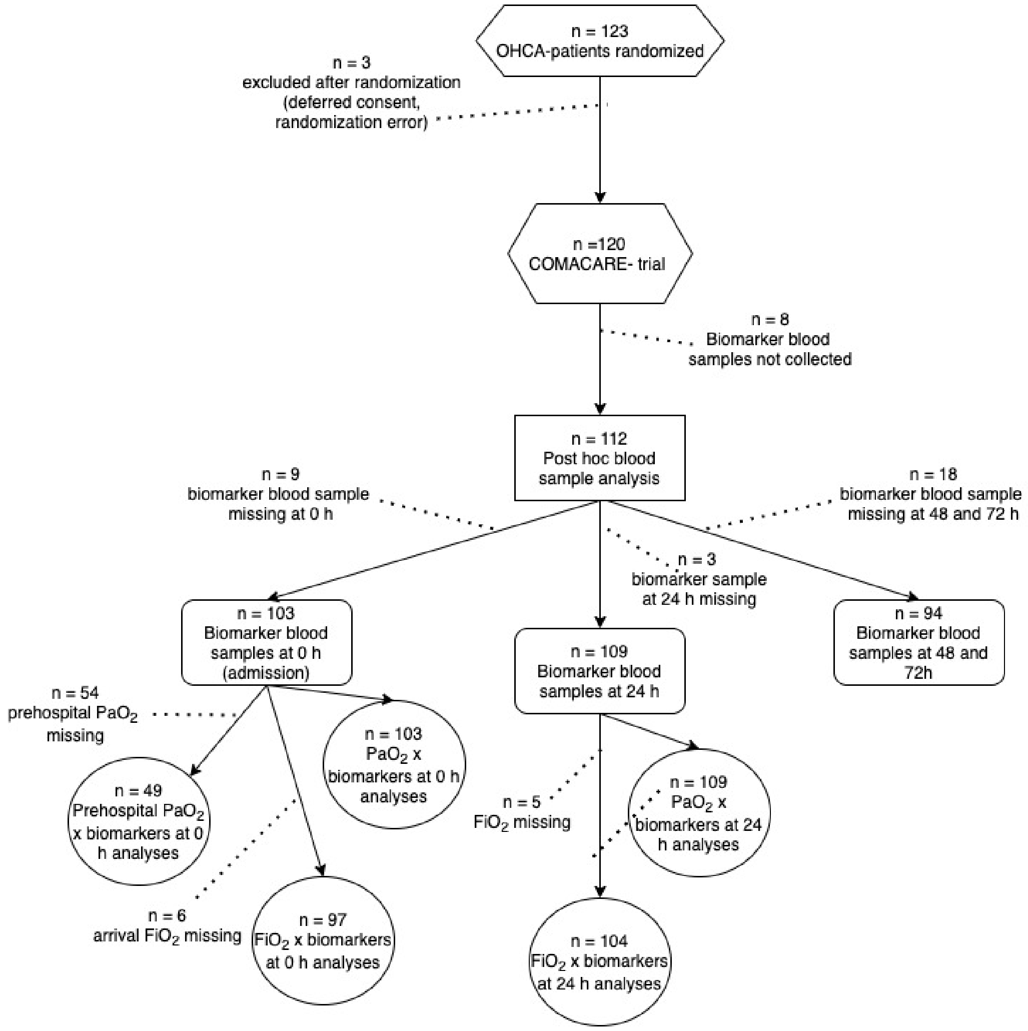

Figure S1. Flow chart of patient inclusion in sub analyses.

Definitions of abbreviations: OHCA= out-of-hospital cardiac arrest.

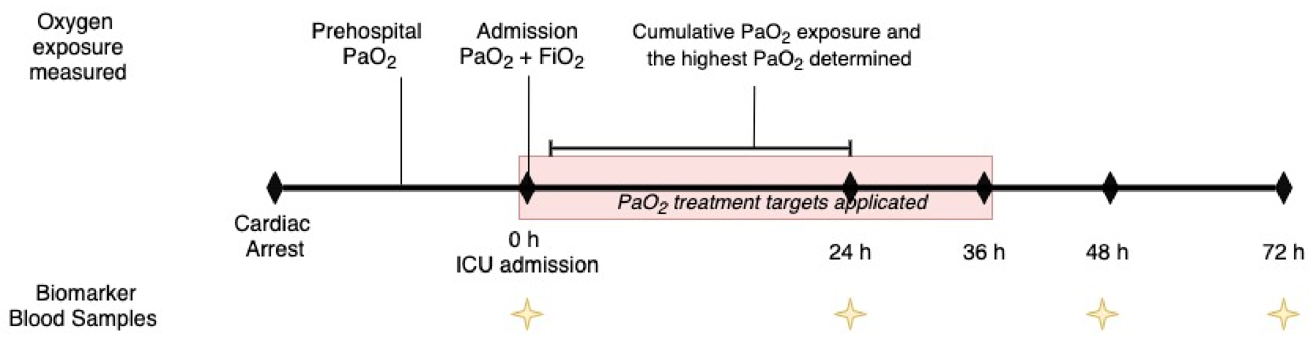

Figure S2. Timeline of the study protocol and blood sample timing.

PaO2 partial pressure of arterial oxygen, FiO2 fraction of inspired oxygen, ICU intensive care unit

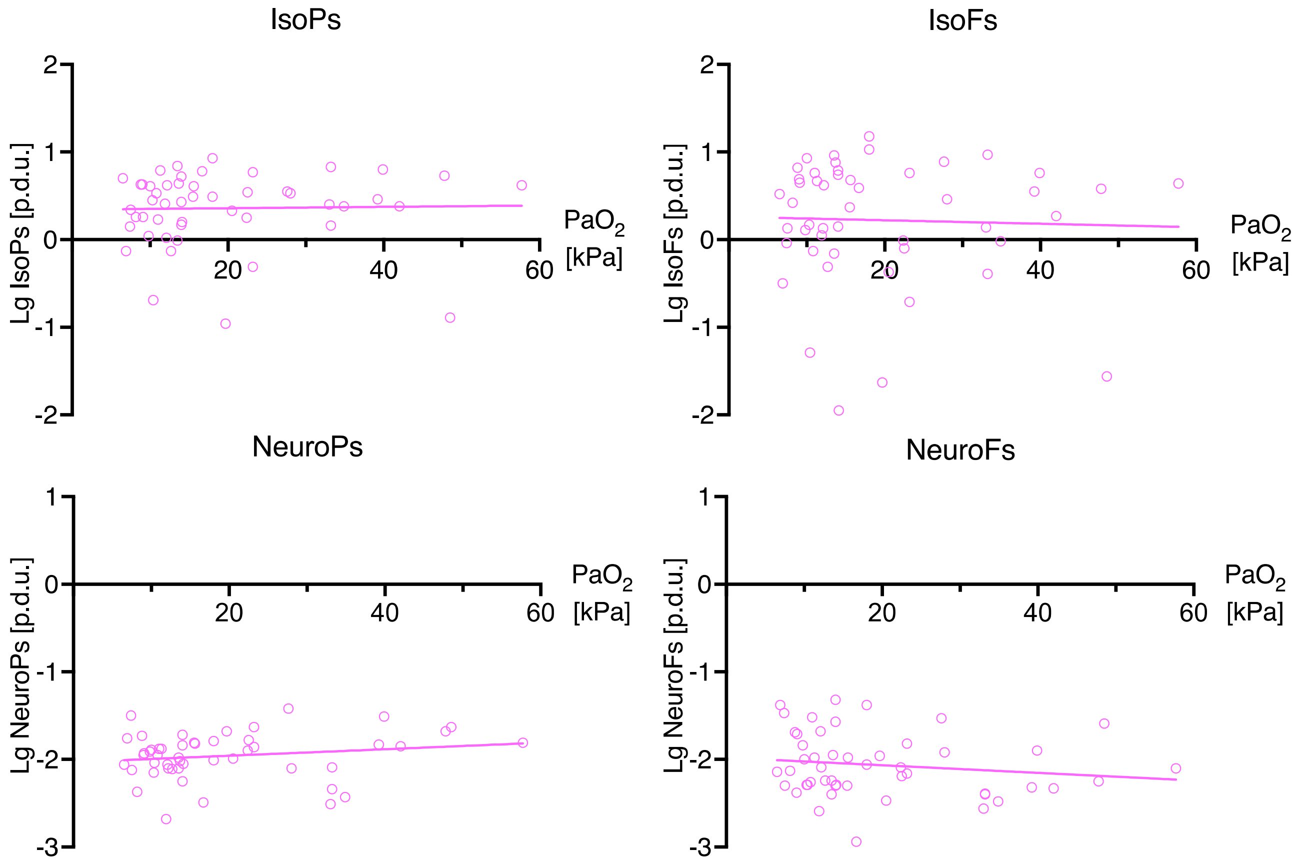

Figure S3. Scatterplot of biomarker levels on ICU admission (0h) and the first prehospital PaO2 determined after return of spontaneous circulation.

Definitions of abbreviations: [p.d.u] = procedure defined unit; Lg = base 10 logarithm

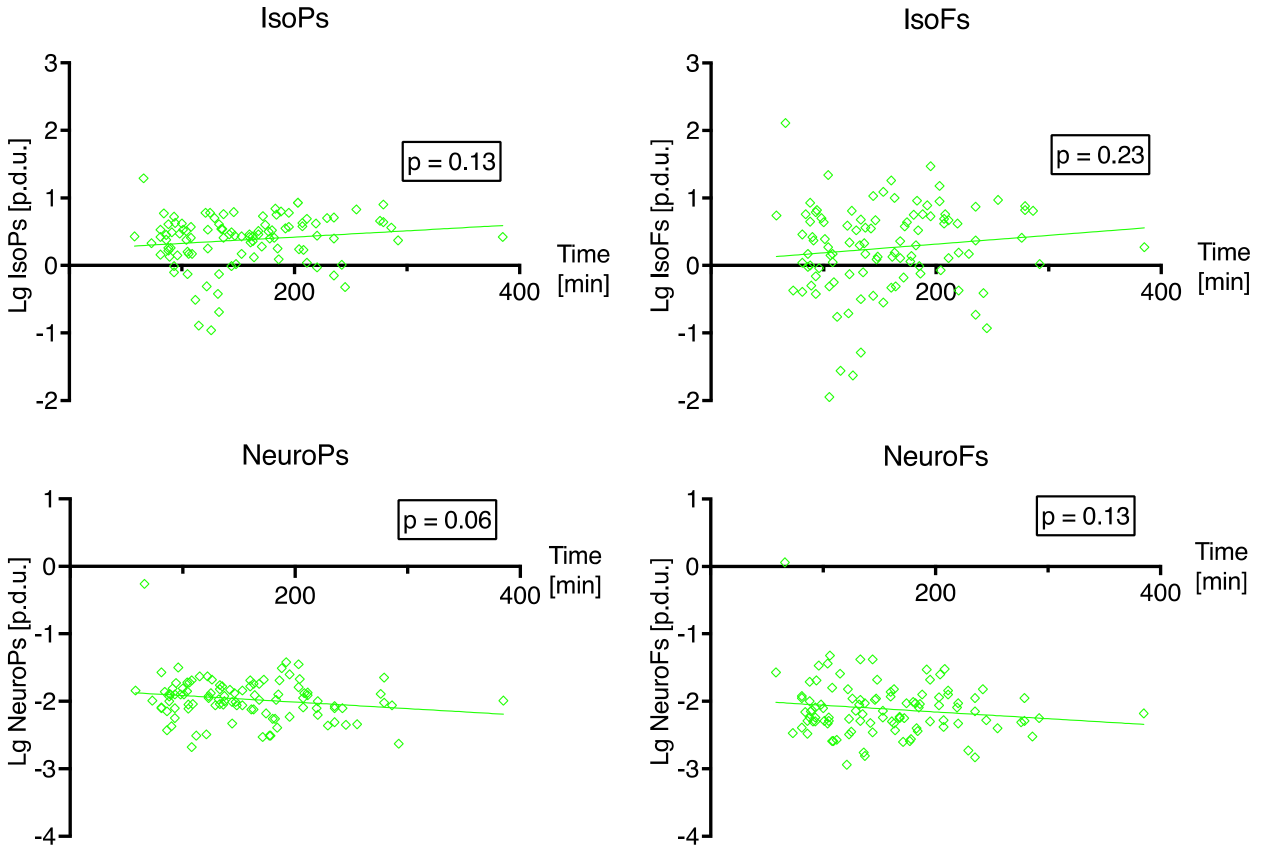

Figure S4. Scatterplot of biomarker levels on admission and time from the arrest to ICU admission.

Linear regression lines summarize the association between variables, p-values are conducted from linear regression analysis. Complete study population is shown in one group.

Definitions of abbreviations: [p.d.u] = procedure defined unit; Lg = base 10 logarithm

Table S1 Comparison of biomarker levels on ICU admission between early vs late arrivers. Early arrivers were defined if the time from cardiac arrest to ICU is below median arrival time (147 minutes).

	**Early arrivers, (CA to ICU delay <147 min)**	**Late arrivers, (CA to ICU delay ≥ 147 min)**	**p-value (Mann-Whitney U)**
	median (IQR)		
IsoPs at 0 h	2.61 (1.43-3.65)	2.90 (1.89 - 4.56)	0.10
IsoFs at 0 h	1.67 (0.52-4.41)	2.37 (1.15-6.49)	0.07
NeuroPs at 0 h, 10e-2	1.23 (0.89 - 1.54)	0.98 (0.63-1.62)	0.17
NeuroFs at 0 h, 10 e-2	0.71 (0.47-1.19)	0.71 (0.47-1.14)	0.94

Definitions of abbreviations: CA= cardiac arrest, ICU= intensive care unit, IQR = interquartile range.

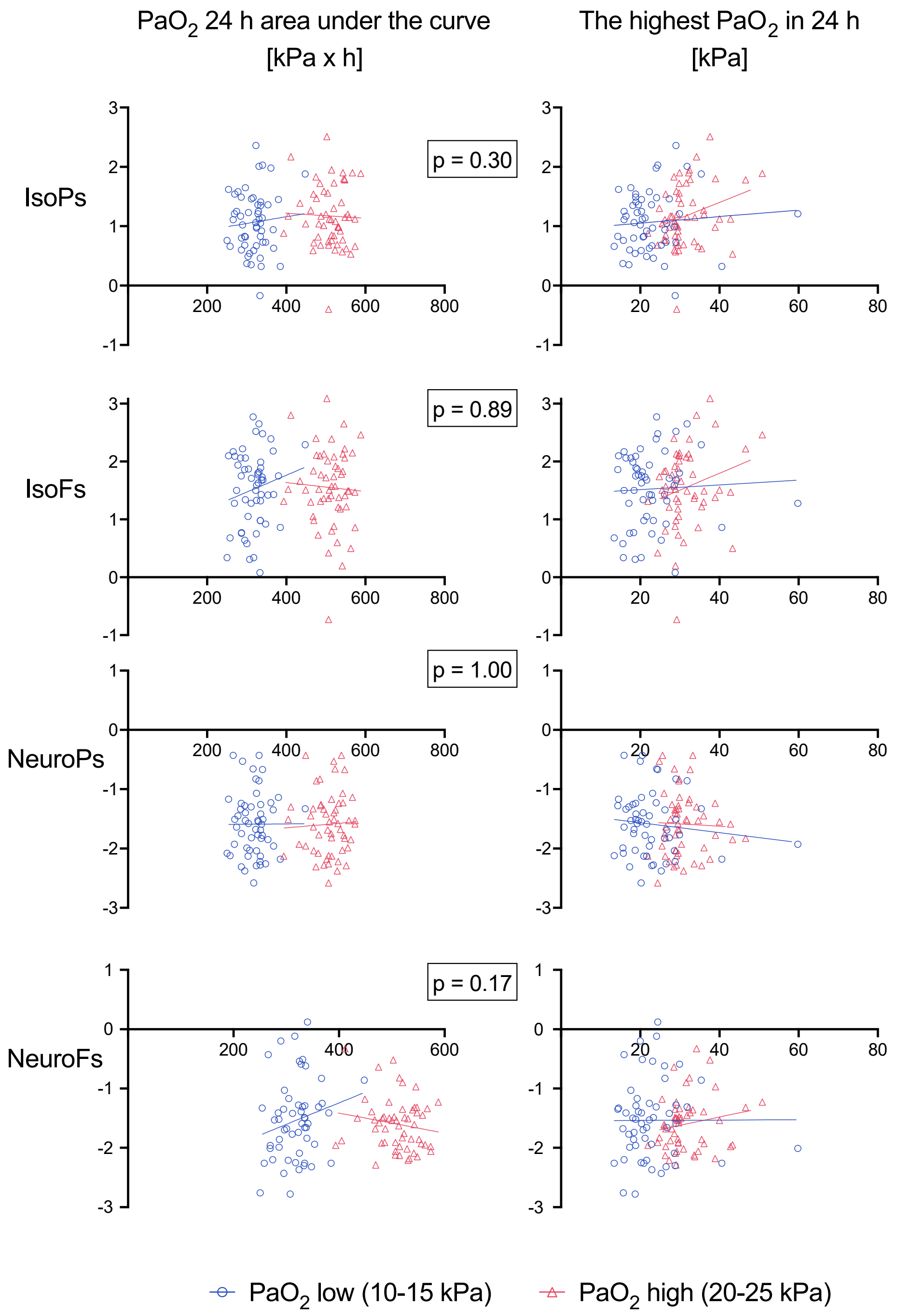

Figure S5. Scatterplot of biomarker levels at 24 h and PaO_2 24 hours area under the curve (left column) and the highest PaO_2 in 24 hours (right column).

Lg transformed biomarker values are presented in the ordinate, and PaO_2 [kPa] and PaO_2 area under the curve [kPa x h] values are presented in the abscissa. P-values indicate significance of Mann Whitney U tests between biomarker levels at 24 h in low vs high PaO_2 groups. Patients are grouped based on PaO_2 treatment targets. Regression line has been drawn through each group, although, there are no actual correlations between the parameters (tiny R^2 values).

Definitions of abbreviations: [p.d.u] = procedure defined unit; Lg = base 10 logarithm ; AUC = area under the curve

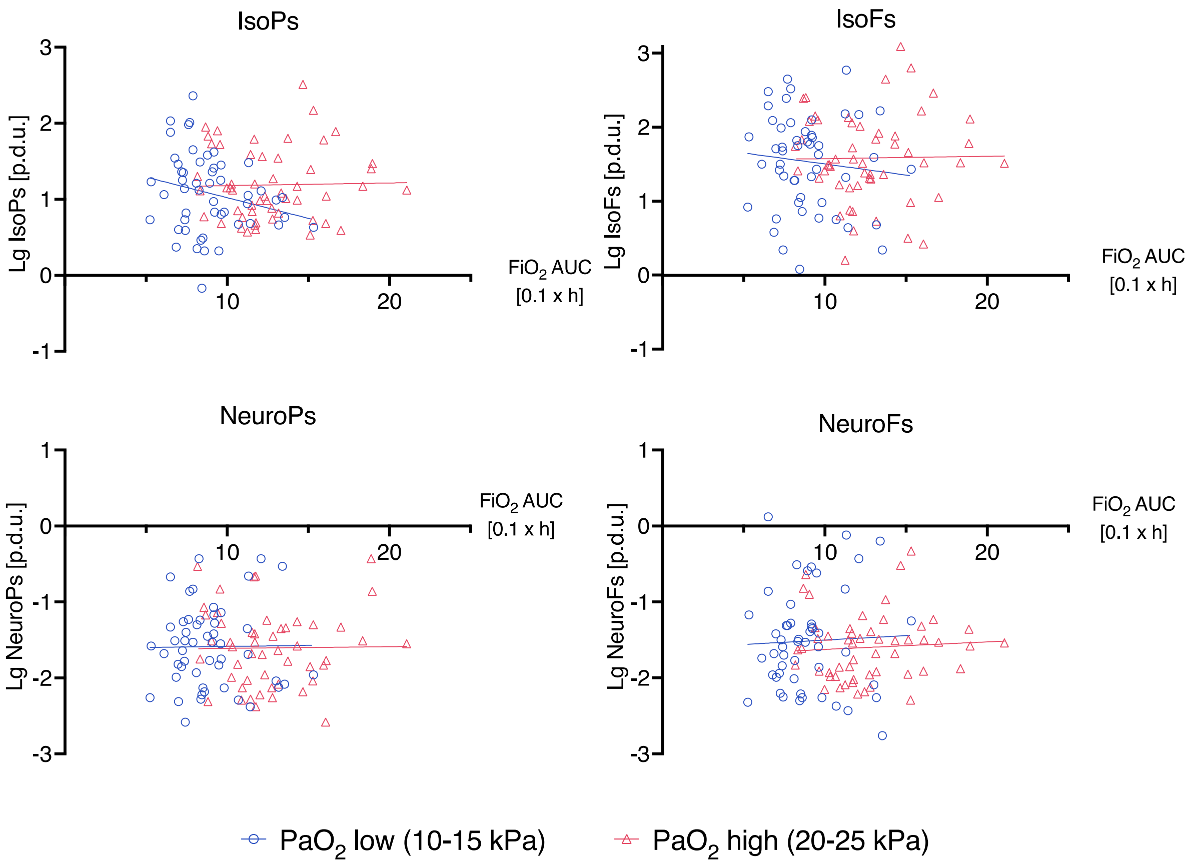

Figure S6. Scatterplot of biomarker levels at 24 h and FiO_2 area under the curve for 24 h.

Patient are grouped based on PaO_2 treatment targets. Regression line has been drawn through each group, although, there are no actual correlations between the parameters (tiny R^2 values).

Definitions of abbreviations: [p.d.u] = procedure defined unit; Lg = base 10 logarithm; AUC = area under the curve

Table S2. Linear mixed model analysis of biomarker levels over time between patients who ended up in good versus bad neurological outcome and between PaO_2 target groups.

	**Time**	**Outcome (CPC 1-2 vs CPC 3-5)**	**Time * Outcome**	**Time**	**PaO_2 (10-15 vs. 20-25 kPa)**	**Time * PaO_2**
**IsoPs**	<0.001	0.24	0.71	<0.001	0.66	0.54
**IsoFs**	<0.001	0.05	0.37	<0.001	0.91	0.53
**NeuroPs**	<0.001	0.043	0.03	<0.001	0.54	0.42
**NeuroFs**	0.16	0.06	0.30	0.32	0.16	0.32

Definitions of abbreviations: CPC =cerebral performance category; [p.d.u] = procedure defined unit; Lg = base 10 logarithm.

Table S3. Comparison of biomarker levels in different time points between patients who ended up in good versus bad neurological outcome at six months after the cardiac arrest.

	**Good outcome CPC 1-2**	**Bad outcome CPC 3-5**	**p-value (Mann-Whitney U)**
Biomarkers			
IsoPs [p.d.u.]			
0 h	3.30 (2.06-5.04)	2.12 (1.18-3.23)	0.006
24 h	14.5 (5.35-39.3)	13.10 (5.74-19.63)	0.69
48 h	7.57 (4.62-18.37)	7.04 (3.94-19.54)	0.64
72 h	3.96 (2.54-6.87)	4.60 (2.76-10.10)	0.17
IsoFs [p.d.u.]			
0 h	2.51 (0.99-5.76)	1.42 (0.54-4.16)	0.08
24 h	47.40 (15.13-120.75)	33.13 (6.16-74.76)	0.88
48 h	16.91 (5.99-56.47)	13.30 (3.42-51.37)	0.98
72 h	4.97 (2.44-14.52)	7.10 (2.37-22.86)	0.31
NeuroPs [p.d.u.]			
0 h	1.11 (0.78-1.62)	1.12 (0.53-1.59)	0.78
24 h	2.73 (1.02-4.50)	2.61 (0.93-5.32)	0.55
48 h	1.57 (0.81-2.95)	1.46 (0.80-3.95)	0.42
72 h	1.02 (0.72-1.51)	1.03 (0.72-3.32)	0.31
NeuroFs [p.d.u.]			
0 h	0.71 (0.50-1.13)	0.69 (0.34-1.05)	0.88
24 h	1.71 (0.79-4.5)	1.67 (0.53-5.38)	0.31
48 h	1.06 (0.82-2.48)	0.82 (0.56-3.32)	0.49
72 h	0.59 (0.39-0.85)	0.69 (0.45-1.28)	0.19

Definitions of abbreviations: CPC =cerebral performance category; [p.d.u] = procedure defined unit; Lg = base 10 logarithm.

Table S4. Linear regression analysis to predict biomarker levels at 0 h.

	**R^2**	**B**	**95% CI for B** lower bound	upper bound	**P-value**
**Lg IsoPs at 0 hours**	0.04				
Propensity score for risk of high admission PaO_2		0.32	-0.13	0.77	0.16
The first PaO_2 (kPa)		0.0003	-0.01	0.01	0.51
FiO_2 upon admission		-0.16	-0.68	0.37	0.56
**Lg IsoFs at 0 hours**	0.03				
Propensity score for risk of high admission PaO_2		0.11	-0.67	0.89	0.77
The first PaO_2 (kPa)		0.01	-0.01	0.03	0.19
FiO_2 upon admission		-0.38	-1.30	0.54	0.41
**Lg NeuroPs at 0 hours**	0.06				
Propensity score for risk of high admission PaO_2		0.001	-0.38	0.38	1.00
The first PaO_2 (kPa)		0.0003	-0.008	0.0009	0.94
FiO_2 upon admission		0.39	-0.06	0.83	0.10

**Lg NeuroFs at 0 hours**	0.07				
Propensity score for risk of high admission PaO_2		-0.07	-0.61	0.32	0.54
The first PaO_2 (kPa)		0.04	-0.01	0.01	0.69
FiO_2 upon admission		0.24	0.09	1.19	0.02

Definitions of abbreviations: [p.d.u] = procedure defined unit; Lg = base 10 logarithm.

References

1. Jakkula, P.; Reinikainen, M.; Hästbacka, J.; Pettilä, V.; Loisa, P.; Karlsson, S.; Laru-Sompa, R.; Bendel, S.; Oksanen, T.; Birkelund, T.; et al. Targeting Low- or High-Normal Carbon Dioxide, Oxygen, and Mean Arterial Pressure After Cardiac Arrest and REsuscitation: Study Protocol for a Randomized Pilot Trial. *Trials* **2017**, *18*, 507, doi:10.1186/s13063-017-2257-0.
2. Sánchez-Illana, Á.; Shah, V.; Piñeiro-Ramos, J.D.; Di Fiore, J.M.; Quintás, G.; Raffay, T.M.; MacFarlane, P.M.; Martin, R.J.; Kuligowski, J. Adrenic Acid Non-Enzymatic Peroxidation Products in Biofluids of Moderate Preterm Infants. *Free Radical Biology and Medicine* **2019**, *142*, 107–112, doi:10.1016/j.freeradbiomed.2019.02.024.
3. Sánchez-Illana, Á.; Thayyil, S.; Montaldo, P.; Jenkins, D.; Quintás, G.; Oger, C.; Galano, J.M.; Vigor, C.; Durand, T.; Vento, M.; et al. Novel Free-Radical Mediated Lipid Peroxidation Biomarkers in Newborn Plasma. *Analytica Chimica Acta* **2017**, *996*, 88–97, doi:10.1016/j.aca.2017.09.026.
4. Cummins, R.O.; Chamberlain, D.A.; Abramson, N.S.; Allen, M.; Baskett, P.J.; Becker, L.; Bossaert, L.; Delooz, H.H.; Dick, W.F.; Eisenberg, M.S.; et al. Recommended Guidelines for Uniform Reporting of Data from Out-of-Hospital Cardiac Arrest: The Utstein Style: A Statement for Health Professionals from a Task Force of the American Heart Association, the European Resuscitation Council, and Heart and Stroke Foundation of Canada, and the Australian Resuscitation Council. *Circulation* **1991**, *84*, 960–975, doi:10.1161/01.CIR.84.2.960.
5. Neuhäuser, M. Wilcoxon–Mann–Whitney Test. In *International Encyclopedia of Statistical Science*; Lovric, M., Ed.; Springer Berlin Heidelberg, **2011**; 1656–1658.
6. McHugh, M.L. The Chi-Square Test of Independence. *Biochemia Medica* **2012**, *23*, 143–149, doi:10.11613/BM.2013.018.
7. Molenberghs, G. Linear Mixed Model - Encyclopedia of Mathematics Available online: http://encyclopediaofmath.org/index.php?title=Linear_mixed_model&oldid=38546 (accessed on 25 May 2021).
8. Duricki, D.A.; Soleman, S.; Moon, L.D.F. Analysis of Longitudinal Data from Animals with Missing Values Using SPSS. *Nature Protocols* **2016**, *11*, 1112–1129, doi:10.1038/nprot.2016.048.
9. Jawień, W. Searching for an Optimal AUC Estimation Method: A Never-Ending Task? *Journal of Pharmacokinetics and Pharmacodynamics* **2014**, *41*, 655–673, doi:10.1007/s10928-014-9392-y.
10. Schober, P.; Vetter, T.R. Linear Regression in Medical Research. *Anesthesia and analgesia* **2021**, *132*, 108–109, doi:10.1213/ANE.0000000000005206.
11. LaValley, M.P. Logistic Regression. *Circulation* 2008, *117*, 2395–2399.
